# Thyroid hormones modifications among COVID-19 patients undergoing pulmonary rehabilitation

**DOI:** 10.3389/fendo.2023.1192561

**Published:** 2023-07-14

**Authors:** Laura Croce, Elisabetta Zampogna, Francesca Coperchini, Pietro Costa, Patrizia Pignatti, Dina Visca, Antonio Spanevello, Mario Rotondi

**Affiliations:** ^1^ Department of Internal Medicine and Therapeutics, University of Pavia, Pavia, Italy; ^2^ Unit of Endocrinology and Metabolism, Laboratory for Endocrine Disruptors, Istituti Clinici Scientifici Maugeri, Istituto di Ricovero e Cura a Carattere Scientifico (IRCCS), Pavia, Italy; ^3^ Respiratory Rehabilitation of the Institute of Tradate, Istituti Clinici Scientifici Maugeri, Istituto di Ricovero e Cura a Carattere Scientifico (IRCCS), Tradate, Italy; ^4^ Allergy and Immunology Unit, Istituti Clinici Scientifici Maugeri, Istituto di Ricovero e Cura a Carattere Scientifico (IRCCS), Pavia, Italy; ^5^ Department of Medicine and Surgery, Respiratory Diseases, University of Insubria, Varese, Italy

**Keywords:** thyroid, COVID-19, pulmonary rehabilitation, non-thyroidal-illness, low-T3 syndrome

## Abstract

**Introduction:**

Patients with severe COVID-19 often experience long-lasting disabilities that can improve after pulmonary rehabilitation. Moreover patients with severe COVID-19 display thyroid function alterations due to a non-thyroidal illness syndrome (NTIS). The aim of our study was to evaluate thyroid function parameters among patients hospitalized for COVID-19 who were eligible or not to respiratory rehabilitation and their modifications during follow-up.

**Materials and methods:**

Post-COVID-19 patients referred to a Respiratory Rehabilitation Unit were evaluated. Outpatients, not candidate for rehabilitation, were enrolled as Control group. Patients who had completed a 4-week-rehabilitation program were enrolled as Rehabilitation Group. All patients were evaluated at T0 (4 weeks after the discharge home in Control Group and after completion of rehabilitation in Rehabilitation Group) and at T1 (3 months after T0).

**Results:**

The final study group included 39 patients (20 in the Rehabilitation group and 19 in the Control group). Patients in the Rehabilitation Group had more frequently received invasive or non-invasive ventilation, had a longer length-of-stay in referring hospitals, had a higher number of comorbidities and displayed a worse performance at 6-minute-walking-test (6MWT) and Short-Physical-Performance-Battery-test (SPPB). FT3 values were lower at T0 in the Rehabilitation Group, while TSH and FT4 values were similar in the two groups. While no significant modifications in thyroid-function-parameters were observed in the Control Group, a significant increase in FT3 value was observed in the Rehabilitation Group at T1. Participants of both groups had improved the results of 6MWT at T1, while SPPB values improved only in the Rehabilitation Group.

**Conclusions:**

COVID-19 patients after pulmonary rehabilitation experience an increase in FT3 values during follow-up, paralleled with an amelioration of functional capabilities.

## Introduction

Coronavirus-Disease-19 (COVID-19) is the disease caused by SARS-CoV-2, characterized by various clinical manifestations, going from mild/asymptomatic forms (in about 81% of infected people), to severe or critical (1). The most typical and frequent symptoms at onset are fever, cough, and shortness of breath. Additional symptoms can include weakness, fatigue, nausea, vomiting, diarrhea and anosmia. A significant percentage of cases requires admission to intensive-care-units (ICU) due to acute respiratory distress syndrome that requires mechanical ventilation support (2). In a subset of patients a severe and life threatening complication, the “Cytokine storm”, can occur, characterized by a fulminant and fatal hyper-cytokinemia associated with multi-organ failure (3, 4). Patients who survive the acute phase of COVID-19 often experience long lasting symptoms and disabilities, including fatigue, dyspnea, muscle weakness and impaired mobility, with a consequent decrease in quality of life (5). In particular, in hospitalized patients without any prior motor limitation recovering from COVID-19, a high prevalence of muscle weakness and physical performance impairment has been observed, especially among those requiring mechanical ventilation, sedation, and prolonged intensive care unit (ICU) stay (6, 7). Rehabilitation intervention following the acute phase of COVID-19 (including positioning and respiratory management, medicine, physiotherapy, and psychological support) can help reduce hospital length of stay and improve patient status and quality of life (8, 9).

Among the several organs possibly affected by COVID-19, also the thyroid was object of extensive study during this pandemic (10). The most agreed upon finding is that patients with COVID-19 can experience a “non-thyroidal illness syndrome”, especially in severe cases (11–16). This syndrome is characterized by a wide spectrum of thyroid function alterations, most commonly a reduction in free tri-iodothyronine (FT3) and thyroxine (FT4) circulating levels, and can have a prognostic significance in critically ill patients (17). In particular, the “cytokine storm” that characterizes the most severe COVID-19 cases can significantly impact of thyroid function and cause a severe non-thyroidal illness (18).

The aim of our study was to evaluate thyroid function parameters among patients hospitalized for COVID-19 who were eligible or not to respiratory rehabilitation and their modifications during follow-up.

## Materials and methods

### Study participants

The study included post-COVID-19 inpatients and outpatients referred to Istituti Clinici Scientifici (ICS) Maugeri, Tradate, Italy between March 13, 2020 and July 31, 2021. The study was approved by the Central Ethics Committee of ICS Maugeri (CEC 2279; March 12, 2020), and patients signed the consent form. Inpatients were transferred from intensive and sub-intensive care units, pneumology units or general wards after SARS-CoV 2 negative test, to perform respiratory rehabilitation. Outpatients, not candidate for rehabilitation, (Control group), were discharged home after hospitalization for acute illness and SARS-CoV 2 negative test and were enrolled for the study at the follow-up visit 4 weeks from the discharge at home (T0). Patients of the Rehabilitation Group were enrolled after completion of the 4-week-rehabilitation program (T0). All patients were re-evaluated 3 months after T0 (T1).

The inclusion criteria were:1) availability of thyroid function parameters measurement (including TSH, FT3, FT4) at baseline (T0) and at the 3 months follow-up visit (T1); 2) availability Short Physical Performance Battery (SPPB) at baseline (T0) and at the 3 months follow-up visit (T1). The exclusion criteria were: 1) presence of pre-existing thyroid diseases 2) ongoing therapy with any drug potentially interfering with thyroid function 3) the presence of any thyroid function parameter outside the normal range at baseline.

The following evaluations were performed: clinical examination and anthropometric assessment. Data regarding length of stay (LoS) before admission for pulmonary rehabilitation, previous treatment for acute respiratory failure (ARF) such as Invasive Mechanical Ventilation (IMV), Non-Invasive mechanical Ventilation (NIV), steroid therapy or oxygen use, presence of pulmonary fibrosis at chest CT and arterial blood gases were collected. In individuals under long-term oxygen therapy, assessment had been performed under oxygen at the usual oxygen inspiratory fraction (FiO2).

The burden of comorbidities was estimated through the Cumulative Illness Rating Scale Comorbidities Index (CIRS-CI) and the Cumulative Illness Rating Scale Severity Index (CIRS-SI) (19). CIRS-CI was calculated assigning to each item a score between 0 (none) and 4 (extremely severe), total score reflecting the mean value of the first 13 items. CIRS-CI was obtained by the sum of the items with score ≥3. Data regarding inflammatory markers, including C-reactive protein (CRP), platelet count and D-dimer were collected.

### Rehabilitation program and functional outcome measures

A multidisciplinary program was applied. Endurance exercise training, strength training involving upper and lower peripheral muscle, individual educational sessions and when necessary, tailored diet and psychological support were included in the 4-weeks inpatient program. Intensity, timing and modality of training were tailored to the individual patient according to age, clinical severity, length of immobilization, comorbidities, starting from a minimum of one, 20 minute daily session up to two/three, 30 minute daily sessions.

The following outcome measures were assessed when allowed by patients’ clinical conditions and safety or organizational issues:

i) The lower extremity function was assessed by means of the Short Physical Performance Battery test (SPPB) (20, 21) with the predicted normal values of Bergland et al. (22). The total SPPB score ranges from 0 to 12: 1–2: severe; 3–8 moderate disability; 9–12 normal.

ii) Exercise tolerance was assessed by the Six minutes Walking Test (6MWT) (23) using the predicted values of Enright et al. (24). The baseline value of patients unable to perform the test was considered as 0 for analysis.

### Serum thyroid function assays

TSH, FT3 and FT4 were measured with the Alinity I system (Abbott Laboratories, IL, USA) which is an automated analyzer that utilizes chemiluminescent microparticle immunoassay (CMIA) principle, by using anti-analyte coated paramagnetic microparticles and anti-analyte acridinium-labeled conjugates. The reaction is measured as relative light units, which have a direct or inverse relationship with the amounts of analyte in the sample.

The intra-assay coefficient of variation (CV) values ranged from 2.7 to 3.8% for FT3, from 2.6 to 3.1% for FT4, from 1.5 to 2.1% for TSH, from 2.3 to 2.8%.

The analytical sensitivities were 1.25 pg/ml for FT3, 0.42 ng/dl for FT4, 0.0083 mIU/l for TSH (third-generation TSH assay). Normal ranges were for TSH 0.35-4.94 µUI/ml, for FT3 1.71-3.71 pg/ml, for FT4 0.70-1.48 ng/dl. Quality control pools at low, normal, and high concentrations for all parameters were present in each assay.

### Statistical analysis

Statistical analysis was performed using the SPSS Software (SPSS, Inc.). Between-groups comparisons were performed using the Student’s t-test for unpaired data and the Mann–Whitney U-test according to a normal or a non-parametric distribution; comparisons were performed using the Student’s t-test for paired data and the Wilcoxon’s test according to a normal or a non-parametric distribution. Frequencies among groups were compared using the χ2-test with Fisher’s correction when appropriate. A *p* value of <0.05 was considered statistically significant. Results are expressed as mean ± SD for normally distributed variables and median and interquartile range (IQR) for non-parametric variables.

## Results

Out of 185 individuals (in and outpatients) post-COVID-19 screened during the study period, 39 patients (20 in the Rehabilitation group and 19 in the Control group) were included in the study. As shown in **Table 1**, the patients in the two groups were similar in terms of age, sex and BMI. Apart from a slightly lower PaO_2_/FiO_2_ ratio among the Rehabilitation Group, no difference in baseline blood gas parameters were observed. The levels of three different markers of inflammation (PCR, D-dimer and platelet count) were similar in the two groups. While a similar percentage of patients had needed oxygen therapy during the acute phase of the infection, a higher percentage of patients in the rehabilitation group had received invasive or non-invasive ventilation. The two Groups were similar in terms of number of patients who had needed steroid therapy in the acute phase of the disease, with a similar cumulative steroid dose. A higher number of patients was still receiving low-dose steroid therapy at T0 in the Rehabilitation Group. In details, the seven patients in the rehabilitation Group were receiving a median prednisone dose of 37.5 mg (IQR 25-37.5) per day, while the only patient in the Control Group receiving steroid therapy was taking 25 mg of prednisone per day. The LoS in referring hospitals for acute COVID-19 was longer in the rehabilitation group. Moreover, patients in the rehabilitation group had a more severe condition and higher number of comorbidities as assessed by the CIRS index. Baseline thyroid function evaluation showed significantly lower FT3 values in the Rehabilitation Group, while TSH and FT4 values were similar in the two groups.

**Table 1:** comparison of clinical and biochemical characteristics of patients included in the Rehabilitation or in the Control Group.

	Rehabilitation (N 20)	Control (N 19)	*P* value
Age (years)	67.2 ± 8.0	60.5 ± 12.3	0.050
Male, n (%)	14 (70.0%)	12 (63.2%)	0.651
BMI	27.5 ± 5.8	29.2 ± 4.4	0.323
Previous IV, n (%)	8 (40.0%)	0 (0%)	0.002
Previous NIV, n (%)	15 (75.0%)	4 (21.1%)	<0.001
Previous O_2_ need, n (%)	17 (85.0%)	13 (68.4%)	0.219
Previous need of steroid therapy during acute COVID-19, n (%)	14 (70.0%)	8 (42.1%)	0.079
Previous cumulative dose of steroid therapy, prednisone equivalents, mg	195.7± 62.4	165.0± 62.1	0.279
Patients requiring low-dose steroid therapy at T0 n (%)	7 (35.0%)	1 (5.3%)	0.022
Pulmonary Fibrosis at Chest CT, n (%)	7 (35.0%)	6 (31.6%)	0.821
LoS in acute hospitals, days	46.4 ± 18.1	29.2 ± 15.2	0.019
PaO_2_/FiO_2_	375.5 ± 57.8 (n=16)	411.2 ± 39.7 (n=17)	0.046
PaO_2_, mm Hg	79.4 ± 11.4	86.3 ± 8.3	0.053
PaCO_2_, mm Hg	36.7 ± 3.6	36.1 ± 2.1	0.508
pH	7.41 ± 0.04	7.41 ± 0.02	0.761
CRP mg/dl	0.47(0.14-0.84)	0.23 (0.16-0.40)	0.262
D-dimer, ng/ml	500 (330–870)	480 (315–620)	0.752
Platelet count (n x 10^9^/L)	243 (190–336)	223 (196-286)	0.820
CIRS-SI, score	1.60 ± 0.24	1.34 ± 0.20	<0.001
CIRS-CI, score	3.40 ± 1.31	1.84 ± 1.46	0.001
TSH*	1.476 (1.120-2.992)	1.346 (0.986-1.687)	0.187
FT3	2.54 ± 0.35	2.83 ± 0.33	0.015
FT4	0.88 ± 0.11	0.95 ± 0.14	0.053

Data are expressed as n (%) or mean ± sd. *data expressed as median (IQR). BMI, body mass index; IV, invasive ventilation; NIV, non-invasive ventilation; O_2_, oxygen; LoS, length of stay; PaO2, arterial oxygen tension; PaCO2, arterial carbon dioxide tension; FiO2, inspired oxygen fraction; CIRS-SI, Cumulative Illness Rating Score Severity Index; CIRS-CI, Cumulative Illness Rating Score Comorbidities Index, TSH, thyrotropin; FT3, free tri-iodothyronine; FT4, free-thyroxine; CRP, C-Reactive Protein.

As shown in **Table 2**, no significant variations between T0 and T1 could be observed in the two groups in the levels of inflammation markers and in blood gas parameters.

**Table 2:** Comparison of blood gases and inflammatory parameters at T0 and T1 in the Rehabilitation and Control Groups.

Rehabilitation Group	T0	T1	*p* value
Blood gases			
PaO_2_/FiO_2_	382.0 ± 56.2	386.0 ± 43.3	0.856
PaO_2_, mm Hg	80.2 ± 11.8	81.8 ± 8.6	0.725
PaCO_2_, mm Hg	36.5 ± 3.6	37.5 ± 3.7	0.327
pH	7.42 ± 0.03	7.41 ± 0.03	0.708
Inflammatory parameters			
CRP mg/dl	0.47 (0.14-0.84)	0.24 (0.10-0.59)	0.110
D-dimer, ng/ml	500 (330-870)	505 (295-638)	0.306
Platelet count (n x 10^9^/L)	243 (190-336)	233 (196-278)	0.446

Control Group	T0	T1	*p* value
Blood gases			
PaO_2_/FiO_2_	411.2 ± 39.7	399.2 ± 33.9	0.304
PaO_2_, mm Hg	86.3 ± 8.3	83.8 ± 7.1	0.303
PaCO_2_, mm Hg	36.1 ± 2.1	37.5 ± 4.1	0.166
pH	7.41 ± 0.02	7.40 ± .02	0.122
Inflammatory parameters			
CRP mg/dl	0.23 (0.16-0.40)	0.15 (0.16-0.26)	0.074
D-dimer, ng/ml	480 (315-620)	355 (315-468)	0.109
Platelet count (n x 10^9^/L)	223 (196-286)	220 (193-267)	0.538

Data are expressed as mean ± SD or median (IQR). PaO2, arterial oxygen tension; PaCO2, arterial carbon dioxide tension; FiO2, inspired oxygen fraction; CRP, C-Reactive Protein.

**Figure 1:**
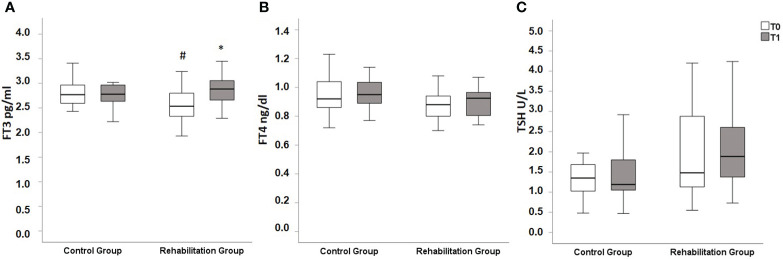
Box plot representing thyroid function parameters at baseline (T0, white bars) and at the 3 months follow-up (T1, grey bars). **(A)**: at T0, FT3 values were significantly higher in Control Group when compared with Rehabilitation Group [2.89 (2.65-3.07) pg/ml in Control Group vs 2.54 (2.32-2.82) pg/ml in Rehabilitation Group (*p*=0.021)]. At T1 FT3 values significantly increased in Rehabilitation Group, reaching a median of 2.89 (2.65-3.07) pg/ml (*p*=0.007 vs T0), while no differences between T0 and T1 could be observed in Control Group [at T1 2.78 (2.62-2.98) pg/ml, *p*=0.672 vs T0]. There were no significant differences between the two groups at T1 (*p*=0.627) **(B)** similar levels of FT4 were observed between the two groups both at T1 [0.92 (0.86-1.07) ng/dl in Control Group vs 0.86 (0.74-0.94) ng/dl in Rehabilitation group, *p*=0.134] and at T2 [0.95 (0.89-1.04) ng/dl in Control Group vs 0.93 (0.75-0.97) ng/dl in Rehabilitation Group, *p*=0.113]. No significant variations between T0 and T1 could be observed either in the Control group (*p*=0.286) nor in the Rehabilitation Group (*p*=0.243) **(C)** similar levels of TSH were observed between the two groups both at T1 [1.35 (0.99-1.69) U/L in Control Group vs 1.48 (1.12-2.99) U/L in Rehabilitation group, *p*=0.189] and at T2 [1.19 (1.02-1.81) U/L in Control Group vs 1.88 (1.34-2.63) U/L, *p*=0.079]. No significant variations between T0 and T1 could be observed neither in the Control group (*p*=0.601) nor in the Rehabilitation Group (*p*=0.502). Values reported as median (IQR). TSH, thyrotropin; FT3, free tri-iodothyronine; FT4, free-thyroxine.*p<0.05 vs T0 (Wilcoxon’s test). ^#^p<0.05 vs Control group at the same time point (Mann–Whitney test).

**Figure 1** shows the comparison between thyroid function parameters evaluated at baseline and those evaluated at the 3 months follow-up in the two groups. While no significant modifications in thyroid function parameters were observed in the Control Group, a significant increase in FT3 value was observed in the Rehabilitation Group.

**Figure 2:**
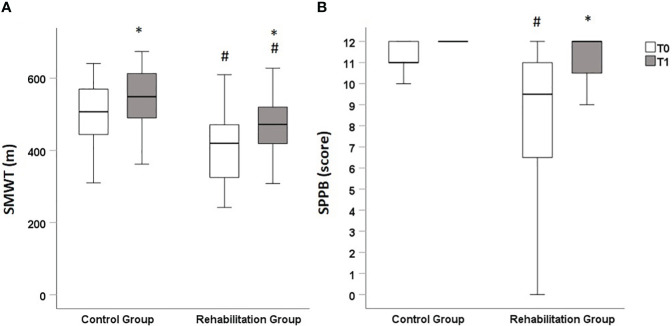
Box plot representing results of functional evaluations at baseline (T0, white bars) and at the 3 months follow-up (T1, grey bars). Panel **(A)**: at T0, 6MWT values were significantly higher in Control Group when compared with Rehabilitation Group [522 (444-570) meters in Control Group vs 405 (303-476) meters in Rehabilitation Group, *p*=0.014]. At T1, 6MWT values significantly increased both in Control Group, reaching a median 549 (480-614) meters (*p*=0.004 vs T0), and in Rehabilitation Group, reaching a median of 485 (419-520) meters (*p*=0.001 vs T0). At T2, 6MWT values were still significantly higher in the Control group than in the Rehabilitation Group (*p*=0.036) Panel **(B)** at T0, SPPB scores were significantly higher in Control Group when compared with Rehabilitation Group [11 (11, 12) points in Control Group vs 10 (6–11) in Rehabilitation Group, *p*=0.014]. At T1, SPPB values significantly increased in Rehabilitation Group, reaching a median of 12 (10–12) points (*p*=0.001 vs T0). No significant modifications in SPPB score were observed in Control group (*p*=0.164), in which all patients had a full score (12) at T2. At T2, SPPB values were similar between the Control group and the Rehabilitation Group (*p*=0.214). Values reported as median (IQR). 6MWT: six-minute-walking test. SPPB: Short Physical Performance Battery test.*p<0.05 vs T0 (Wilcoxon’s test). ^#^p<0.05 vs Control group at the same time point (Mann–Whitney test).

**Figure 2** shows the baseline and follow-up values of SPPB and 6MWT. The results show that at baseline both SPPB and 6MWT were significantly higher in the Control Group, suggesting a better performance among these patients. After 3 months, participants of both group had improved the results of the 6MWT, but remained significantly lower in the Rehabilitation Group, while SPPB values improved only in the Rehabilitation Group. No significant variations in terms of

## Discussion

The results of the present study show that patients with post-critical COVID-19 experience an increase in FT3 levels at a 3 months follow-up time after completing a respiratory rehabilitation program. This improvement is paralleled by an increase in lower extremity strength and exercise tolerance, as testified by an increase in 6MWT and SPPB. We observed no significant modifications in thyroid function parameters in the group of COVID-19 who were not eligible for rehabilitation. It should be noted that the patients included in the rehabilitation group were characterized by lower FT3 at the beginning of the study. This is not surprising, since these patients were characterized by a worse respiratory performance (as testified by the lower PaO_2_/FiO_2_ ratio), by a higher percentage of patients who had required both invasive and non-invasive ventilation and by a higher burden of comorbidities. These findings are in line with previous results on post COVID-19 subjects (11–16). Several studies have showed that the alterations in thyroid function typical of the Non-thyroidal illness syndrome occur frequently among COVID-19 patients and are more pronounced among those with a more severe illness and requiring mechanical ventilation (11–16). Our results show that the improvement of the general conditions after pulmonary rehabilitation of COVID-19 is reflected also in an increase in FT3 levels and a reverting of the Non-Thyroidal-Illness Syndrome. Since this improvement is observed at the 3 months visit after the conclusion of rehabilitation program, we cannot exclude that part of this phenomenon may be due to a general health recovery not directly linked with the rehabilitation intervention. This concept would be supported by the finding that an increase in 6MWT occurred also in patients not undergoing rehabilitation. Indeed, a previous similar study performed among patients undergoing rehabilitation for critical neurological conditions showed an increase in FT3 values in the early stages of rehabilitation (25). In our case, the lack of an evaluation of thyroid function parameters at the moment of acute illness does not allow us to evaluate the effect of early rehabilitation. Nevertheless, the fact that an improvement in FT3 values is observed several months after the acute phases of COVID-19 suggests that these patients can still experience a clinical improvement even in the chronic phases of the disease.

This study has several limitations, mainly due to the limited number of patients included. Nevertheless, the availability of a complete thyroid function evaluation and the exclusion of patients with pre-existing thyroid condition strengthens our results.

In conclusion, COVID-19 patients who have undergone pulmonary rehabilitation experience an increase in FT3 values during follow-up, paralleled with an amelioration of functional capabilities. Prospective studies including a higher number of patients are needed to confirm these promising preliminary results.

## Data availability statement

The raw data supporting the conclusions of this article will be made available by the authors, without undue reservation.

## Ethics statement

The studies involving human participants were reviewed and approved by Central Ethics Committee of ICS Maugeri (CEC 2279; March 12, 2020). The patients/participants provided their written informed consent to participate in this study.

## Author contributions

LC, EZ, AS and MR contributed to conception and design of the study. EZ and LC organized the database. LC performed the statistical analysis. LC wrote the first draft of the manuscript. EZ, PC and PP wrote sections of the manuscript. All authors contributed to the article and approved the submitted version.
